# Moderate threat causes longer lasting disruption to processing in anxious individuals

**DOI:** 10.3389/fnhum.2014.00626

**Published:** 2014-08-19

**Authors:** Sophie Forster, Anwar O. Nunez-Elizalde, Elizabeth Castle, Sonia J. Bishop

**Affiliations:** University of California, Berkeley, Berkeley, CA, USA

**Keywords:** attention, distraction, threat, anterior cingulate cortex (ACC), fusiform gyrus, anxiety, amygdala

## Abstract

Anxiety is associated with increased attentional capture by threat. Previous studies have used simultaneous or briefly separated (<1 s) presentation of threat distractors and target stimuli. Here, we tested the hypothesis that high trait anxious participants would show a longer time window within which distractors cause disruption to subsequent task processing, and that this would particularly be observed for stimuli of moderate or ambiguous threat value. A novel temporally separated emotional distractor task was used. Face or house distractors were presented for 250 ms at short (∼1.6 s) or long (∼3 s) intervals prior to a letter string comprising Xs or Ns. Trait anxiety was associated with slowed identification of letter strings presented at long intervals after face distractors with part surprise/part fear expressions. In other words, these distractors had an impact on high anxious individuals’ speed of target identification seconds after their offset. This was associated with increased activity in the fusiform gyrus and amygdala and reduced dorsal anterior cingulate recruitment. This pattern of activity may reflect impoverished recruitment of reactive control mechanisms to damp down stimulus-specific processing in subcortical and higher visual regions. These findings have implications for understanding how threat-related attentional biases in anxiety may lead to dysfunction in everyday settings where stimuli of moderate, potentially ambiguous, threat value such as those used here are fairly common, and where attentional disruption lasting several seconds may have a profound impact.

## INTRODUCTION

Effects of threat-related distractors on competition for attentional resources have been reported using spatial, object-based, and temporal manipulations of attention (see Bar-Haim et al., 2007; Bishop, 2008 for reviews). These effects are largest in anxious individuals and have been argued to play a role in the maintenance, and possibility even etiology, of anxiety (MacLeod and Mathews, 2012). Evolutionarily, it may be advantageous to rapidly allocate processing resources to threatening stimuli when these are encountered. However, it may be equally important to be able to speedily evaluate weak or ambiguous threat cues as of little immediate concern and return to the task in hand. A key factor in determining whether threat-related attentional capture causes disruption in daily life is likely to be the speed with which attentional resources are made re-available for task-related processing after such stimuli are encountered. In understanding anxiety-related functional impairments, it may therefore be particularly important to consider the time course over which attentional capture by threat cues is resolved. If anxious individuals are slower to damp down neural responses to weak or uncertain threat cues, this could result in prolonged disruption to task performance as a result of ongoing competition for processing resources from such stimuli. The present study sought to test this possibility by examining anxiety-related interference from distractors of intermediate, potentially ambiguous threat value over a longer time course than has previously been considered.

To date, the issue of whether threat related stimuli can produce interference that lasts for several seconds has received little research attention. Rather, interference from task-irrelevant threat distractors has primarily been explored in tasks where distractor and task-related stimuli occur simultaneously (e.g., Richards and Millwood, 1989; Vuilleumier et al., 2001; Bishop et al., 2004a, 2007; Lim and Pessoa, 2008), where targets (i.e., the stimulus that participants are asked to respond to) occur prior to distractors (Dolcos and McCarthy, 2006) or where targets follow distractors by under 1000 ms (e.g., MacLeod and Mathews, 1988; Reed and Derryberry, 1995; Most et al., 2005). The latter category includes studies examining spatial attentional engagement and disengagement by means of cuing tasks such as the “dot probe” task (e.g., Reed and Derryberry, 1995; Fox et al., 2001) and studies examining temporal interference using the “emotional blink” variant of the attentional blink (e.g., Most et al., 2005). Such short-lived interference appears unlikely to significantly disrupt daily life.

Studies of attentional disruption from threat-related distractors, as described above, have also typically used stimuli which clearly signal threat – such as images of violence scenes or mutilated bodies from the International Affective Picture System (IAPS; Bradley and Lang, 2007) and faces showing strong expressions of fear from sets such as the picture of facial affect (POFA; Ekman and Friesen, 1976). Such stimuli are encountered relatively rarely in daily life, and may indeed signal a threat demanding an urgent response. In contrast, weaker threat cues are encountered more frequently, with milder expressions of shock or apprehension potentially signaling anything from plates being dropped in a restaurant to a rat running across the sidewalk. It has been proposed that the strength of threat signal needed to capture attention is lower for high anxious individuals, with the result that while relatively strong threat cues are required to capture the attention of low anxious individuals, even relatively weak threat stimuli can capture the attention of high anxious individuals (Mogg and Bradley, 1998). High anxious individuals have also been reported to be more likely to treat emotionally ambiguous stimuli that can be interpreted as either neutral or negative as threat-related than individuals with low levels of anxiety (Eysenck et al., 1987; Mathews et al., 1989). These observations are likely related, with relatively weak threat cues, such as mildly shocked expressions, often being more ambiguous or uncertain in the information they convey and more open to interpretation than stronger threat cues (see Whalen, 2007; Grupe and Nitschke, 2013, for discussions of the centrality that should be given to ambiguity and uncertainty in rethinking the function of “threat-evaluation” circuitry). A central aim of our current study was therefore to investigate whether high trait anxious individuals take longer to evaluate and dismiss relatively weak, potentially ambiguous, threat cues as being of little immediate concern, and return to the task in hand.

A second key aim of our study was to elucidate differences in regional brain function that might underlie prolonged disruption of task-related processing by distractors of milder, more ambiguous, threat value. Previous work has indicated that the extent to which non-emotional distractor stimuli (scenes) disrupt processing of targets varies inversely with “top-down” suppression of distractor-related activity in stimulus specific regions (Gazzaley et al., 2005). Similar interacting influences of top down control mechanisms and “bottom-up” circuitry involved in threat evaluation and distractor representation have also been revealed when emotional distractors are presented concurrently with target stimuli (Bishop et al., 2007). Here, anxious individuals showed both increased distractor-related activity in the amygdala and cortical stimulus-selective regions together with impoverished recruitment of frontal control circuitry. While this work did not examine the temporal duration of distractor-related activity, other recent findings suggest that negative affect is characterized by slower amygdala recovery from threat cues, as opposed to differences in the initial magnitude of amygdala response (Siegle et al., 2002; Schuyler et al., 2014). Here, disruption to subsequent task processing was not examined. The present study extends this previous body of work to consider milder, potentially more ambiguous, threat cues and to examine their impact upon subsequent processing of task-relevant stimuli. Specifically, we explore the possibility that prolonged disruption from such distractors might be associated with both a prolonged stimulus specific response, and impoverished recruitment of reactive control mechanisms, and that this might be most notable in high trait anxious individuals.

In the current study, as in much prior work in the field (e.g., Vuilleumier et al., 2001; Pessoa et al., 2002; Bishop et al., 2004a,b, 2007), we use emotional faces as distractor stimuli. These provide biologically relevant stimuli of varying threat value, where much is already known about the brain mechanisms involved in their processing. “Stimulus specific” brain regions activated by emotional facial expressions include both the fusiform face area (FFA; Kanwisher et al., 1997) and amygdala. The FFA is responsive to faces relative to other classes of stimuli such as houses or letters. FFA activation is modulated by facial expression, being greater for expressions with high than low threat values – e.g., for fearful versus neutral faces (Vuilleumier et al., 2001). It has been argued that this increased FFA signal for faces showing threat-related expressions might be driven by input from the amygdala (Vuilleumier et al., 2001). The amygdala is responsive to emotionally charged faces and has been variously held to play a role in threat evaluation, salience detection, and resolution of ambiguity of potential threat stimuli (Whalen, 1998, 2007). Prolonged processing of mild or ambiguous threat-related expressions might hence be influenced positively by the extent of amygdala and FFA response and negatively by the extent to which frontal control regions are brought online to inhibit the processing of these stimuli.

As alluded to above, the recruitment of frontal attentional control mechanisms has been demonstrated to be disrupted in anxiety (Bishop et al., 2004a; Bishop, 2009; Ansari and Derakshan, 2011) with evidence suggesting that this top-down deficit may interact with bottom-up responsivity to threat cues to determine attentional capture by threat (Bishop, 2007). Distinct brain circuits have been identified that support proactive control (engagement of control processes ahead of time, e.g., prior to anticipated processing competition) and reactive control (online augmentation of control, e.g., to inhibit processing of a given stimulus). Reactive control may be particularly relevant for terminating ongoing processing of distractors when task-relevant stimuli are encountered. Engaging in this form of control is held to activate the dorsal region of the anterior (mid) cingulate cortex (dACC; Braver et al., 2007). In line with this, we have recently reported that activation of this region predicts attentional task performance when reactive control is needed, with high trait anxious individuals showing impoverished recruitment of this region, and slower task performance under these conditions (Forster et al., 2013).

In the current study, we used a temporally separated emotional distractor task in order to test the prediction that when moderate, potentially ambiguous, threat cues are used, anxiety-related differences in disruption to subsequent task-related processing will be observable up to several seconds later. Specifically, we aimed to examine whether slowed recovery from presentation of faces showing surprise/fear blends would be linked to prolonged interference with subsequent letter string identification in high trait anxious individuals. Both short (∼1.6 s) and long (∼3 s) distractor-target intervals were used. The intervals were chosen such that the longer ones would fall within the “recovery” period of distractor-evoked activity based on the data from Schuyler et al. (2014) while the shorter ones would fall more within the initial “responsivity” window. In order to promote reactive (versus proactive) regulation of the distractor response we avoided including very short distractor-target ISIs (<500 ms) of the duration equivalent to those in emotional blink or dot probe studies.

The hypotheses tested were as follows. First that anxiety-related distractor interference effects (i.e., slowing of letter string response times following the presentation of a given distractor type) would be most apparent following faces providing weaker/more ambiguous threat signals (i.e., 66% surprise, 33% fear faces) as opposed to stronger/less ambiguous threat signals (100% fear faces). Second, these differences were expected to be particularly apparent at longer distractor-target ISIs (∼3 s), corresponding to the “recovery” – as opposed to initial “reactivity” – stage described by Schuyler et al. (2014). Third, we tested the hypothesis that lasting interference from weaker, potentially ambiguous, threat stimuli at the longer ISIs would be accompanied by (i) elevated activity in regions involved in threat evaluation and distractor representation (amygdala and FFA, respectively) together with (ii) impoverished recruitment of regions implicated in reactive control (dACC). With regards to (i), prior findings have shown that stimulus specific activity in higher visual cortical regions mediates the influence of amygdala activity on the processing of visual threat-related targets (Lim et al., 2009). Hence, we also explored whether FFA, as opposed to amygdala activity, was most directly linked to individual variability in the impact of face distractors upon subsequent letter string identification.

## MATERIALS AND METHODS

### PARTICIPANTS

Twenty-two participants (15 females, 7 males) aged 18–28 years (*M* = 22 years) performed the Temporally Separated Emotional Distractor task while fMRI data were acquired. Informed consent was obtained prior to participation and the study was conducted under ethical approval from the UC Berkeley Institutional Review Board. All participants were right handed with normal or corrected to normal vision, and no history of psychiatric illness, neurological disease, or head injury. None of the participants were currently taking psychotropic medication. Scores on the Spielberger state-trait anxiety inventory (STAI; Spielberger et al., 1983), collected prior to the scanning session, ranged from 25 to 64 (*M* = 42, SD = 11). These scores are similar to the published norms for this age group (*M* = 36, SD = 10; Spielberger et al., 1983). The behavioral data of one participant was not fully recorded and excluded from all analyses.

### TASK

**FIGURE 1:**
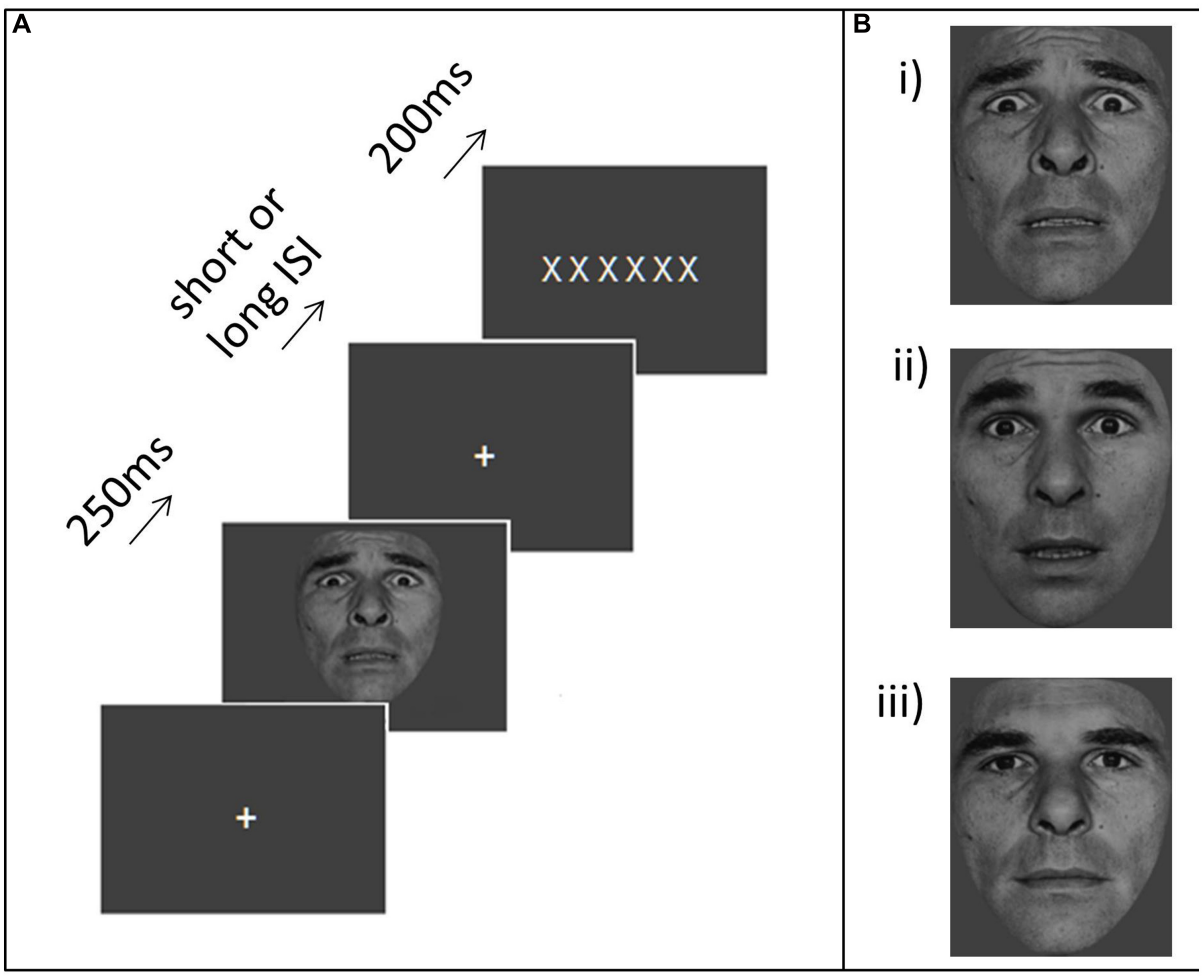
**Task and stimuli. (A)** An example trial. Participants were asked to make speeded forced choice responses indicating whether a letter string comprised Xs or Ns. An irrelevant distractor – either a neutral face, moderate threat face (66% surprise, 33% fear) or strong threat face (100% fear) or a house – was presented for 250 ms at either a short (1.3 or 1.9 s) or long (2.6 or 3.3 s) interval before the letter string display. **(B)** Example face distractors (i) strong threat, (ii) moderate threat, (iii) neutral.

The task used here was adapted from the low perceptual load condition of the emotional distractor task reported by Bishop et al. (2007). In the original task, participants had to identify letters superimposed on a distractor face that varied in emotional expression (fear or neutral) from trial to trial. In the experiment reported here, distractor stimuli were presented prior to, rather than concurrently with, the letter strings. On each trial, a central white fixation cross was presented on a gray background. Following a variable interval (1.3–3.3 s, *M* = 2.3 s), a single distractor image (greyscale, subtending 3∘–3.4∘ horizontally by 2.8∘–3.6∘ vertically) was presented at fixation for 250 ms (**Figure 1A**). Distractors belonged to one of four categories: “strong threat” faces, “moderate threat” faces, neutral faces and houses. The “strong threat” face distractors comprised photographs of four individuals with fearful expressions taken from the POFA set (Ekman and Friesen, 1976). We note our use of the term “strong” to describe the threat level associated these faces is intended relative to other facial expressions rather than across stimulus categories in general, and may be less strong than other forms of threat stimuli (e.g., the threat of electric shock). Nevertheless, these expressions are of strong fear, of an extent unlikely to be encountered in day-to-day life. More moderate and naturalistic expressions, akin to apprehension or shock, were created by morphing between the fear (33%) and surprise (67%) expressions of the same individuals. Ratings (*n* = 21) on a Likert-type scale ranging from 1 (not negative) to 5 (very negative) indicated that the “strong threat” (100% fear) faces were perceived as significantly more negative than the “moderate threat” (morph) faces (mean negativity rating = 3.8 versus 2.5 respectively, *t*(20) = 11.06, *p* < 0.001). Intermediate surprise/fear faces are also more ambiguous or uncertain in their information content (unpublished work from our lab indicates that high trait anxious individuals do not differ from low trait anxious in their categorization of 100% surprise and 100% fear faces but show a sharper transition from “surprise” to “fear” responses when asked to categorize faces that vary in 16.7% morph steps between these two “end” points). The “neutral” faces used were morphed between neutral (83.3%) and happy (16.7%) expressions of the same individuals. This was conducted since the 100% neutral faces from the POFA set are often perceived as slightly negative (Lee et al., 2008). The house distractors comprised four images of houses (taken from Bishop et al., 2004a). Example face stimuli are shown in **Figure 1B**.

The distractor stimulus remained onscreen for 250 ms. The fixation cross was then re-presented for a short (1.3 or 1.9 s) or long (2.6 or 3.3 s) interval, followed by a string of six white letters, each subtending 0.5∘ vertically by 0.4∘ horizontally (with the whole string subtending 3∘ horizontally). This letter string was presented for 200 ms. It comprised all Xs or all Ns. Participants were instructed to respond as fast as possible while maintaining accuracy, pressing the key under the index finger for Xs and the key under the middle finger for Ns. They were also told that they would see images appearing between the letter target displays, and that they should simply view these, maintaining focus throughout on fixation. The distractor-target intervals were chosen such that the longer ones would lead target presentation to fall within the “recovery” period of distractor-evoked activity based on the data from Schuyler et al. (2014) this was determined to begin about 2 s after the peak brain response to stimulus presentation with negative affect related differences being apparent from ∼2.5 s after the initial peak of the BOLD response to distractor presentation. The shorter ISIs fall toward the end of the initial “responsivity” window. Here, an additional constraint was that both ISIs should be sufficiently long to avoid encouraging any proactive control strategy associated with the expectancy of targets appearing very shortly after distractors, hence we did not include very short distractor-target ISIs (<500 ms) of the duration equivalent to those in emotional blink studies. Very short ISIs would also have introduced greater colinearity between regressors into our fMRI analyses due to the slow nature of the BOLD response. Participants performed two runs of 80 trials. In both runs, each category of distractor occurred with equal frequency, in a pseudo-random order (with the constraint that distractors of the same category could not appear more than four times in succession).

### IMAGE ACQUISITION

Blood oxygenation level dependent (BOLD) contrast functional images were acquired with echo-planar T2^∗^-weighted (EPI) imaging using a Siemens Tim Trio 3T magnetic resonance imaging system with a 12 channel head coil. Each image volume comprised 25 3mm thick slices (interslice gap: 0.75 mm; inplane resolution: 2.4 × 2.4 mm; flip angle: 74∘; echo time: 0.54 ms; bandwidth: 2126 Hz; repetition time, TR: 2.0 s; TE: 35 ms). Slice acquisition was descending and axial oblique, angled to avoid the eyeballs. Data were acquired in two scanning runs of approximately 7 min. The first five volumes of each run were discarded to allow for T1 equilibration effects. These acquisition parameters were chosen to minimize voxel size while covering all the brain regions of interest (ROIs). In some subjects, cerebellum and part of motor cortex was not covered by our slice prescription. In order to aid co-registration, an additional eight EPI volumes were acquired using the same parameters as the task data but with an increased number of slices and adjusted TR.

### IMAGE ANALYSIS

Data were analyzed using SPM5 (Wellcome Department of Imaging Neuroscience, London, UK). After conversion from DICOM to NIfTI format, diagnostics were run on the time series for each imaging run. Following an approach similar to that adopted by Carp (2011) and Power et al. (2012), bad volumes (with unusually high changes in mean whole brain signal intensity), were replaced by interpolation of the volumes on either side. In line with findings by Power et al. (2012), these bad volumes tend to correspond to those with notable spikes in movement. Regressors were created to model out the (replaced) volumes in the final analysis. Subsequent to this initial data-cleaning step, image realignment (correcting for head movement) was conducted, followed by slice time correction. The subject’s T1 was aligned to their EPI data using SPM 5′s default coregistration routines. Following this, the T1 was transformed into standard (MNI) space and the transformation applied to the EPI images. For the T1 to MNI transformation we used SPM 5′s combined segmentation and normalization procedure which iteratively combines segmentation of the structural image (into gray matter, white matter, and CSF), spatial normalization and bias correction using both an initial affine transformation and subsequent non-linear deformation of tissue probability maps (Ashburner and Friston, 2005). Images were resampled into MNI space with 2 mm isotropic voxels and smoothed with a Gaussian kernel of 8 mm full-width half-maximum (FWHM).

General linear modeling of the BOLD data was conducted using SPM 5. Onsets for each of the four distractor types (strong threat faces, moderate threat faces, neutral faces, houses), and for letter strings were modeled as a function of distractor-target ISI (short or long) by delta functions convolved with the canonical hemodynamic response function (HRF), giving 10 regressors of interest. Low-frequency drifts were removed using a high-pass filter (180 s). In order to examine the timecourse of BOLD responses to the distractors, we also conducted a finite impulse response (FIR) analysis (Glover, 1999; Goutte et al., 2000; Ollinger et al., 2001). The latter model allowed us to isolate activation occurring earlier or later than that captured by the peak of the canonical HRF. Specifically, we were interested in interrogating ACC activity at around or slightly prior to the time of the target presentation – this equated most closely to time bin 3 for the short ISI and time bin 4 for the long ISI. For completeness, additional analyses were conducted using bins 2–4 for all ROIs (see Supplementary Tables S1 and S2; also see Figure S1 for ROIs and Figure S2 for visualization of FIR timecourses).

In both the canonical HRF and FIR models, nuisance regressors were included in order to reduce task-unrelated variance (noise). These comprised six realignment (movement) regressors, regressors indicating volumes where “bad scans” had been replaced by interpolation of neighboring volumes, and mean time-series extracted from white matter and outside of brain masks.

#### Region of interest analyses

The MARSBAR toolbox (Brett et al., 2002) was used to extract the mean activity associated with each task regressor from pre-defined ROIs. These ROIs were selected to index activation to the distractor images and “top-down” reactive control of this activity. Face responsive ROIs comprised the FFA (Kanwisher et al., 1997) and the amygdala, the latter being particularly responsive to faces with fearful expressions (Morris et al., 1996; Whalen et al., 1998; Vuilleumier et al., 2001) and implicated, more broadly, in threat evaluation and ambiguity resolution (Whalen, 1998, 2007). The FFA ROIs were 8 mm spheres centered on peak activations taken from task data described in Bishop et al. (2004b), MNI co-ordinates, right: 42, -52, -20; left: -40, -50, -18. The amygdala ROIs were taken from the MNI automated anatomical labeling (AAL) atlas. A ROI was also created for dorsal ACC. Previous findings have implicated this region in reactive control, including the inhibition of processing of, and response to, non-target stimuli (Braver et al., 2007; Aron, 2011). The dACC ROI was a 10 mm radius sphere centered on MNI coordinates: 0, 30, 21. This central co-ordinate was derived from a meta-analysis of cognitive control tasks (Duncan and Owen, 2000) as described previously (Bishop et al., 2008; Forster et al., 2013). All ROIs are shown in Figure S1 in Supplementary Material. We note that we chose to use these *a priori* defined ROIs as opposed to using a functional localiser or orthogonal contrasts from current task data to define ROIs on a subject by subject basis. We believe this approach has benefits for individual difference research of this nature where variations in hyper- and hypo-activation of regions could impact ease of ROI definition between subjects and vary systematically with measure of interest (anxiety). We also note, that we use ROIs adopted in prior work by our group, to allow comparability across studies and to be clear their selection was not *post hoc*. Finally, as we have strong *a priori* hypotheses regarding opposing patterns of modulation by trait anxiety of activity in dACC versus FFA and amygdala (with effects of anxiety upon FFA and amygdala most probably reflecting common, not independent, modulatory influences), that are driven by our previous work (Bishop, 2007; Bishop et al., 2007), and limit our investigations to just these regions, we do not apply corrections for comparisons across ROIs. In our reporting of our results, one-tailed tests are used where there is a clear directional hypothesis. Two-tailed tests are used in all other cases. Most typically this is for comparisons included for completeness, but where no difference between conditions was predicted by our hypotheses. In addition all hierarchical regression analyses are reported using two-tailed tests, as per convention.

An initial analysis was conducted to check for habituation effects in the amygdala, given previous reports suggesting this might be observed across trials (Breiter et al., 1996). This analysis found no differences in the amygdala activation to strong threat (compared to neutral or moderate threat faces, or houses) between the two runs (all *p*’s > 0.2, two-tailed). Hence, all reported analyses were conducted using data from both runs. Due to the relative nature of the BOLD signal, and the absence of a good implicit baseline for our conditions of interest, all fMRI data analyses reported are based on contrasts between two or more task conditions.

## RESULTS

### CROSS-GROUP SUMMARY DETAILS OF PARTICIPANT PERFORMANCE

Error rates were low as anticipated. Performance indices (both letter string identification reaction times and error rates) are presented as a function of the distractor type to precede the letter string in **Table 1**.

**Table 1:** Mean (SE in parentheses) reaction time (RT) and percentage error rate as a function of distractor type and ISI.

		Strong threat face	Moderate threat face	Neutral face	House
RT	Short ISI	549 (19)	543 (19)	538 (14)	543 (12)
	Long ISI	543 (19)	532 (15)	543 (17)	535 (16)
% Error	Short ISI	6.82 (1.81)	8.81 (2.29)	7.86 (2.35)	7.61 (2.30)
	Long ISI	6.19 (1.54)	6.46 (1.62)	6.90 (1.94)	6.67 (1.90)

### TRAIT ANXIETY LINKED TO SLOWED LETTER IDENTIFICATION AT LONG (∼3 S) INTERVALS AFTER DISTRACTORS OF MODERATE THREAT VALUE

**FIGURE 2:**
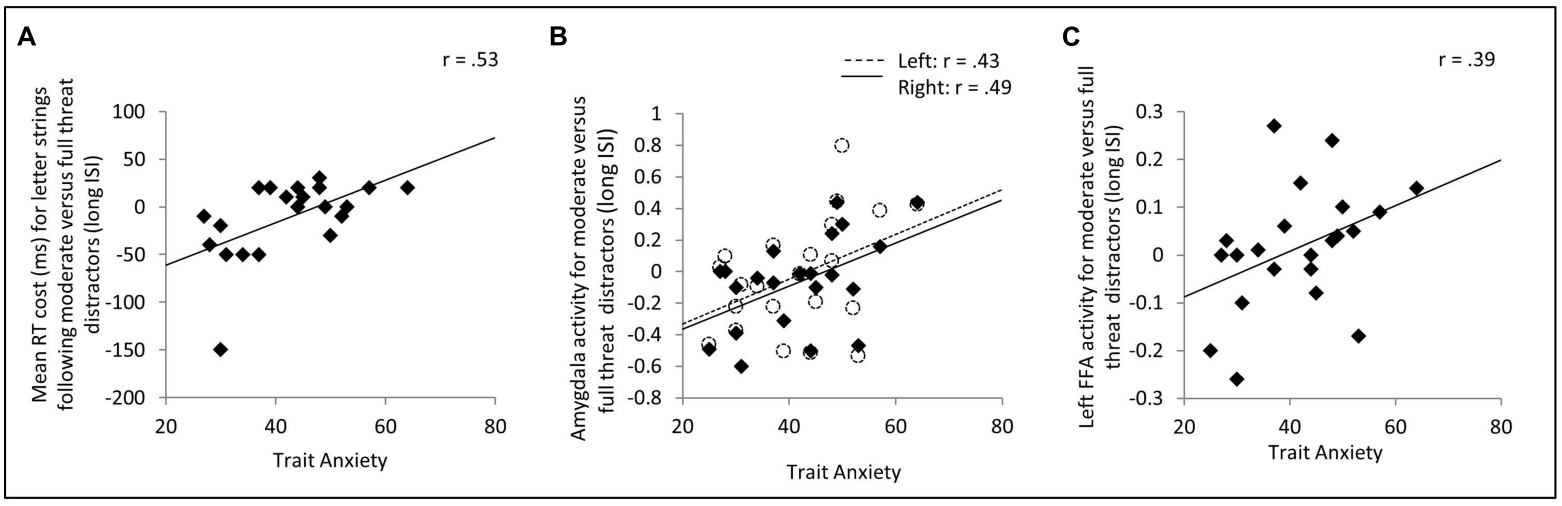
**Effects of trait anxiety upon target identification reaction time (RT) and distractor-related activity in the amygdala and FFA.** **(A)** On trials with long (2.6 or 3.3 s) distractor-target intervals, trait anxiety was positively correlated with RT slowing for letter strings following moderate threat versus full threat distractor faces. **(B,C)** Elevated trait anxiety was associated with both increased amygdala **(B)** and increased FFA **(C)** activity to moderate threat versus full threat distractor faces. Note. Removal of the outlier in **(A)** had a negligible effect on the correlation, *r*(18) = 0.52, *p* < 0.01, one-tailed. In **(B)** and **(C)**, activity refers to mean % blood oxygen level dependent (BOLD) signal change across the region of interest in question. This was modeled using a canonical hemodynamic response function. FFA, fusiform face area.

We first examined the predicted relationship between trait anxiety and interference with target processing from moderate threat distractor faces, especially at long distractor-target ISIs. Correlation analyses revealed a significant positive relationship between trait anxiety and letter identification slowing following moderate versus strong threat face distractors at long distractor-target ISIs, *r*(19) = 0.53, *p* = 0.007, one-tailed (**Figure 2A**). There was also a near-significant trend, at long distractor-target ISIs, for trait anxiety to be positively associated with letter identification slowing following moderate threat distractor faces versus neutral distractor faces, *r*(19) = 0.35, *p* = 0.059, one-tailed. However, no such relationship was observed between trait anxiety and letter identification RT slowing following fearful versus neutral faces at long ISIs (*p* = 0.43, two-tailed), nor were there any significant correlations between anxiety and any RT contrast at short ISIs (*p*’s > 0.8, two-tailed).

The reported anxiety-related differences in target identification RTs, at long ISIs following moderate versus strong threat distractors, could either primarily reflect high trait anxious individuals showing slowing following moderate threat distractors (as predicted) or low trait anxious individuals showing slowing following strong threat distractors. The trend-level positive relationship between trait anxiety and RT slowing, at long ISIs, after moderate threat versus neutral face distractors and absence of any significant relationship between trait anxiety and RT slowing after strong threat versus neutral face distractors supports the interpretation that trait anxiety is linked to slowed resolution of processing of moderate threat value distractors, and associated longer lasting unavailability of resources for task-related processing. We conducted three additional hierarchical regression analyses to further explore this. In these analyses, letter identification RTs, on long ISI trials, was entered separately for each class of distractor as a potential predictor of trait anxiety levels. This enabled us to examine the relative contribution of each task condition to the correlations reported above. In each model, mean letter string identification RTs following neutral faces were included in the first step of the regression model (in order to act as a baseline controlling for non-specific between participant differences in letter identification RTs). Letter identification RTs following neutral face distractors did not predict anxiety (*p* = 0.48, **Table 2**). In model 1, letter identification RTs for strings following moderate and strong threat face distractors were included on step 2. In model 2, only letter identification RTs for strings following moderate threat face distractors were included on step 2 and in model 3, only letter identification RTs for strings following strong threat face distractors were included on step 3. As can be seen from the results presented in **Table 2**, the best fitting model was model 1, with letter identification RTs following moderate threat faces entering as a positive predictor of anxiety and letter identification RTs following strong threat faces entered as a negative predictor of anxiety. The results from models 2 and 3 indicate letter identification RTs following moderate threat distractors had a stronger stand alone relationship with trait anxiety than letter identification RTs following strong threat distractors. Hence, at the long ISI, high trait anxiety was associated with increased interference from moderate threat faces occurring prior to target letter strings, this being clearest when considered relative to the presentation of strong threat face distractors.

**Table 2:** Hierarchical regression analyses examining prediction of trait anxiety by target identification RTs as a function of prior distractor type (long ISI trials only).

		Predictors	β	*p*	*r*^2^ for model	Adj. *r*^2^	*p* for model
Model 1	Step 1	Neutral face	0.021	0.48	0.027	-0.024	0.48
	Step 2	Neutral face	0.064	0.22	0.597	0.526	0.001*
		Moderate threat face	0.231	0.0002*		
		Strong threat face	-0.207	0.001*			
Model 2	Step 1	Neutral face	0.021	0.48	0.027	-0.024	0.48
	Step 2	Neutral face	-0.071	0.15	0.26	0.18	0.068^†^
		Moderate threat face	0.124	0.029*			
Model 3	Step 1	Neutral face	0.021	0.48	0.027	-0.024	0.48
	Step 2	Neutral face	0.079	0.30	0.064	-0.040	0.55
		Strong threat face	-0.056	0.41			

p = two-tailed.

*Significant at *p* < 0.05 two-tailed.

*^†^Significant at *p* < 0.05 one-tailed (*a priori* hypothesis).*

Consistent with the correlation analysis results reported earlier, a regression model equivalent to model 1 but for short ISI trials did not significantly predict trait anxiety levels (*p*’s > 0.3 for the model and for all predictors).

Finally, we note that, across participants, there were no consistent patterns of differences in letter identification RT as a function of the nature of the preceding distractor at either distractor-target ISI (*p*’s > 0.2, two-tailed). Rather, differences in the extent to which particular distractor types produced RT interference emerged as a function of trait anxiety.

### TRAIT ANXIETY MODULATES REGIONAL BRAIN ACTIVITY AS A FUNCTION OF FACE DISTRACTOR TYPE

Examining activity in our ROIs, using a canonical HRF based model, revealed that across participants, face distractors were associated with greater FFA and amygdala activity than house distractors [left FFA, *t*(21) = 3.84, *p* = 0.001; right FFA, *t*(21) = 7.00, *p* < 0.001; left amygdala, *t*(21) = 2.65, *p* = 0.015; right amygdala, *t*(21) = 2.34, *p* = 0.029]. Investigation of ROI activity as a function of face distractor type revealed no significant differences in FFA, amygdala or dACC activity either across ISIs or at either distractor-face ISI considered separately (*p*’s > 0.1). This parallels the absence of any cross-group behavioral (RT) interference effects as a function of distractor face type (see section above). Differences in regional brain activity as a function of distractor face type did however emerge as a function of trait anxiety, in line with our behavioral interference findings. Specifically, elevated trait anxiety was associated with increased amygdala activity to moderate versus strong threat distractor faces at long distractor-target ISIs, right amygdala, *r*(20) = 0.49, *p* = 0.010, one-tailed, left amygdala, *r*(20) = 0.43, *p* = 0.024, one-tailed, **Figure 2B**. This relationship was not observed at short distractor-target ISIs (*p*’s > 0.3, two-tailed). There were no other significant anxiety-related differences in amygdala activity as a function of facial expression (i.e., either moderate or strong threat versus neutral) at either ISI (*p*’s > 0.25, two-tailed). In parallel to the amygdala findings, trait anxiety was also positively associated with left FFA activity to moderate versus strong threat face distractors at long ISIs, *r*(20) = 0.39, *p* = 0.036, one-tailed (**Figure 2C**), but was not significantly associated with FFA activity to moderate versus strong threat distractors at short ISIs (*p*’s > 0.7, two-tailed). Also, as for the amygdala, no other anxiety-related differences in FFA activity as a function of facial expression were observed at either ISI (*p*’s > 0.32, two-tailed).

**FIGURE 3:**
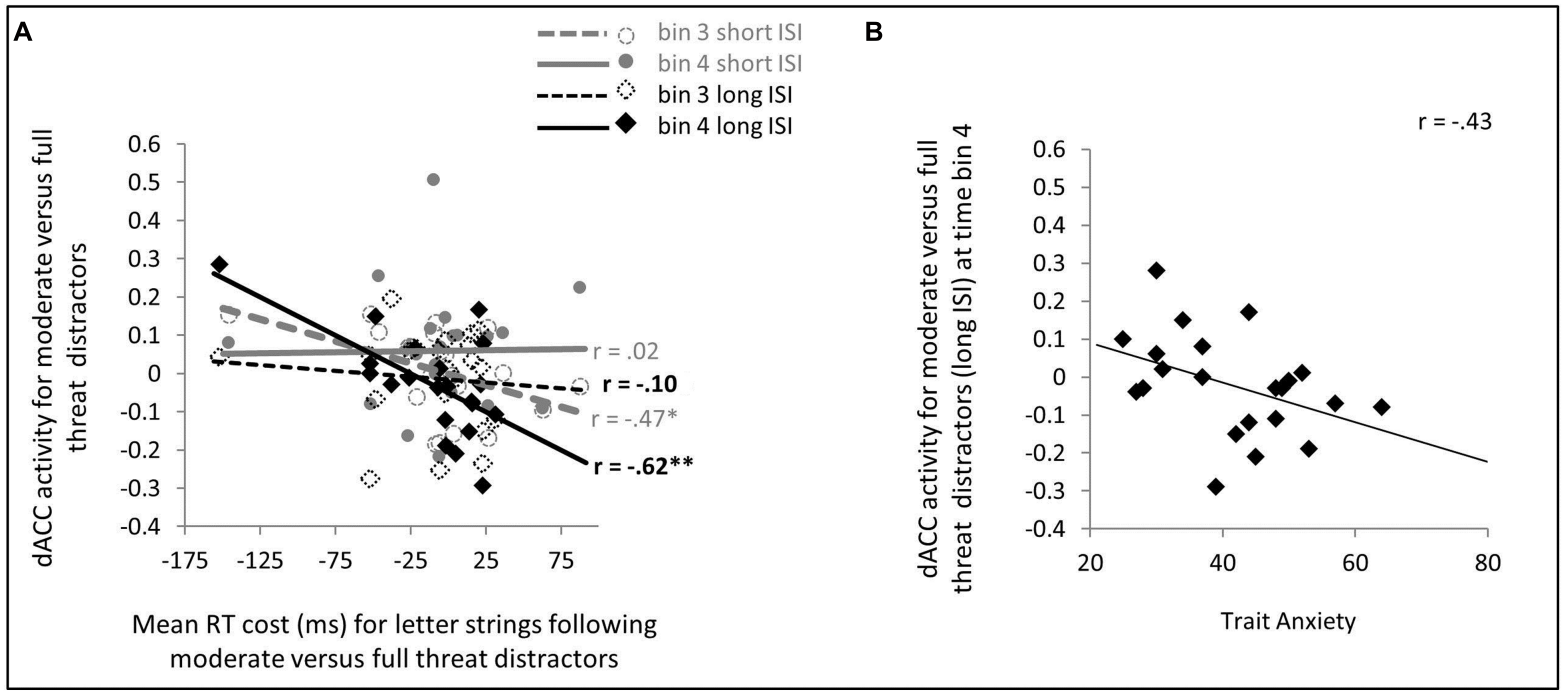
**Dorsal ACC (dACC) activity to moderate versus strong threat distractors: relationship with speed of letter target identification **(A)** and trait anxiety **(B)**.** **(A)** A finite impulse response (FIR) model was used to extract dACC activity from time bins 3 and 4 (4–6 and 6–8 s, respectively) post distractor presentation. For short distractor-target ISI trials, time bin 3 activity may reflect neuronal activity at a time just prior to, through to concurrent with, letter string onset; whereas for long distractor-target ISIs, this is more likely to be captured by time bin 4 activity. In line with this, on short distractor-target ISI trials, greater time bin 3 dACC activity was significantly associated with faster letter target identification following moderate versus strong threat distractors, whereas no such relationship was observed for time bin 4 dACC activity. This relationship reversed for long distractor-target ISI trials, here time bin 4 dACC activity, but not time bin 3 dACC activity, was significantly linked to speed of letter target identification following moderate versus strong threat distractors. **(B)** On long distractor-target ISI trials, trait anxiety was negatively associated with dACC activity to moderate versus full threat distractors at a point (FIR time bin 4) when competition between ongoing face distractor representation and preparation for, or initiation of response to, letter targets was likely to be maximal. No parallel relationship was observed for short distractor-target ISI trials.

Contrary to expectation, no significant effect of trait anxiety was observed upon dACC activity as a function of distractor facial expression or ISI (*p*’s > 0.1, two-tailed). It is possible, however, that trait anxiety related differences in dACC involvement in facilitating recovery from distractors and reallocation of resources to task-related stimuli might have been missed in the analyses reported above due to their reliance on a simple canonical HRF model to assess activity linked to distractor occurrence. To investigate this, we conducted a FIR analysis of activity following presentation of moderate threat versus strong threat distractors. For the long distractor-target ISI, the 4th time bin (6–8 s after distractor onset) was of particular interest, being most likely to capture distractor-related neuronal activity at around or slightly prior to letter-string (target) onset. In contrast, for short distractor-target ISI trials, activity at around or slightly prior to letter-string (target) onset was more likely to be captured by time bin 3 (4–6 s after distractor onset). In line with this, for long distractor-target ISI trials, dACC activity from time bin 4 was linked to faster detection of targets following moderate versus strong threat distractors, *r*(19) = -0.62, *p* = 0.001, one-tailed, while no such relationship was observed for time bin 3 activity, *r*(19) = -0.10, *p* = 0.66, two-tailed, see **Figure 3A**. For short distractor-target ISI trials, the reverse pattern was observed. Here, dACC activity from time bin 3 was associated with faster detection of letter targets following moderate versus strong threat distractors, *r*(19) = -0.47, *p* = 0.016, one-tailed, no such relationship being observed for time bin 4 activity, *r*(19) = 0.02, *p* = 0.94 two-tailed, **Figure 3A**.

Turning to the key issue – that of the modulatory effects of trait anxiety – effects of trait anxiety on letter detection speed following moderate versus strong threat distractors were only observed for long distractor-target ISI trials, as reported earlier. Consistent with this, a relationship between trait anxiety and dACC activity at the time critical for determining competition between the distractor and letter target was observed for long but not short distractor-target ISI trials. Specifically, for long distractor-target ISI moderate versus strong threat distractor trials, dACC activation at time bin 4 post distractor onset was negatively correlated with trait anxiety, *r*(20) = -0.43, *p* = 0.024, one-tailed (**Figure 3B**). No significant relationship was observed between trait anxiety and time bin 3 activity for these trials or between trait anxiety and either time bin 3 or time bin 4 activity for short distractor-target ISI moderate versus strong threat distractor trials (*p*’s > 0.2, two-tailed).

### ANXIETY-RELATED SLOWING OF TARGET IDENTIFICATION AT LONG INTERVALS FOLLOWING MODERATE THREAT FACE DISTRACTORS MEDIATED BY OPPOSING ACTIVITY PATTERNS IN FFA AND dACC

In light of our initial hypotheses, the next question of interest was whether dACC (with its proposed role in reactive control) and either amygdala or FFA activity (thought to reflect determination of distractors’ threat-relevance and ongoing maintenance of their representation, respectively) would have opposing influences on between participant differences in speed of target detection following moderate versus strong threat distractors. To address this, we conducted a hierarchical regression analysis focusing first on long ISI moderate versus strong threat distractors trials, and including our existing (canonical HRF model) indices of amygdala and FFA activity together with FIR time bin 4 dACC activity as potential predictors of differential slowing of letter detection following moderate versus strong threat distractors. Stepwise entry was used, i.e., predictors were entered one a time, starting with the one that explained the maximum variance in scores on the dependent variable. Further predictors were only entered if they led to a significant increase in explained variance. This revealed that both the left (canonical) FFA response (+ve) and FIR time bin 4 dACC response (-ve) were independent and additive predictors of RT interference following moderate versus strong threat distractor faces at long distractor-target ISIs (**Table 3**). These two brain activity predictors also jointly mediated the relationship between anxiety and RT interference, Sobel test, *z* = -1.97, *p* = 0.025, one-tailed. Complementary findings from regression analyses using (i) a forced entry model and (ii) FIR activation indices for all ROIs are reported within the Supplementary Materials (see Supplementary Data and Tables S1, S2).

**Table 3:** Step-wise regression analyses examining brain activity predictors of letter identification slowing (reaction time cost) following moderate versus strong threat distractors.

Predictors	β	*p*	*r*^2^ for model	Adj. *r*^2^	*p* for model
**(A) Long distractor-target ISI trials**		
Left FFA	0.55	0.001	0.67	0.63	<0.001
dACC (FIR time bin 4)	-0.48	0.003			
**(B) Short distractor-target ISI trials**
dACC (FIR time bin 3)	-0.47	0.033	0.22	0.18	<0.05

*p = two-tailed.*

*These analyses used canonical HRF model FFA and amygdala activation indices and FIR model dACC activation indices. Parallel regression analyses using FIR indices for all regions of interest gave similar results (see Table S1 in Supplementary Materials). FIR, finite impulse response; dACC, dorsal anterior cingulate cortex; FFA, fusiform face area. Time bin 3 = 4–6 s post distractor onset. Time bin 4 = 6–8 s post distractor onset.*

Interestingly, our analyses did not reveal a direct role for amygdala responsivity in mediating the relationship between trait anxiety and slowed letter identification following moderate versus strong threat distractor faces at long distractor-target ISIs. However, there was a significant relationship, across participants, between the amygdala response to moderate versus strong threat distractor faces, on long ISI trials, and the left FFA response: right amygdala, *r*(20) = 0.49, *p* = 0.011, one-tailed; left amygdala, *r*(20) = 0.41, *p* = 0.028, one-tailed. This is consistent with suggestions by Lim et al. (2009) that the amygdala might act to modulate activity in stimulus specific regions (in their case parahippocampal cortex, in our case FFA), activity in the latter most directly influencing task performance. We note that similar amygdala-FFA correlations were also obtained using FIR activation indices (see Supplementary Materials).

Although anxiety-related differences in brain activity and target detection speed were observed only on long distractor-target ISI trials, for completeness an additional stepwise hierarchical regression was conducted to examine predictors of between participant differences in RT slowing after moderate versus strong threat distractor faces at short distractor-target ISIs. Here, canonical bilateral FFA and amygdala activation indices were entered together with FIR time bin 3 dACC activity. As reported earlier, greater time bin 3 dACC activity was linked to faster letter identification following moderate versus strong threat distractor faces, *r*(19) = -0.47, *p* = 0.016, one-tailed (**Figure 3A** and **Table 3**). Neither FFA nor amygdala indices additionally entered as predictors of letter detection time for short ISI moderate versus strong threat distractor trials. In addition, the observed relationship between dACC activity and the time taken by participants to identify targets following moderate versus strong threat distractors on short ISI trials was neither associated with, nor modulated by, differences in trait anxiety (*p*’s > 0.1). This finding was also supported by regression analyses using FIR activation indices for all ROIs (see Tables S1 and S2 in Supplementary Materials).

## DISCUSSION

The findings reported here indicate that trait anxiety is associated with prolonged attentional interference from stimuli of moderate, potentially ambiguous, threat value. Specifically, the presentation of faces with part surprise, part fear expressions lead to slowed responding in high trait anxious individuals when simple target identification was required several seconds after distractor offset. This slowing was mediated by greater distractor-related activity in stimulus specific regions (i.e., FFA), combined with reduced dACC recruitment. Amygdala activity to these distractors did not directly predict anxiety-related RT slowing but was strongly correlated with the extent of FFA activity.

Anxiety-related biases in the processing of stimuli of moderate, or ambiguous, threat value have been reported previously (Mogg and Bradley, 1998). However, to our knowledge, our findings are the first to indicate that slowed resolution of processing of such cues can disrupt task-related processing in high trait anxious participants even several seconds after their offset. Our results are in keeping not only with the suggestion that high and low anxious individuals differ primarily in their response to stimuli of moderate, or ambiguous, threat value (Mogg and Bradley, 1998), but also with the suggestion that individuals high in negative trait affect primarily show impoverished recovery from, rather than altered initial response to, threat-related stimuli (Schuyler et al., 2014).

Turning to consider the brain mechanisms involved, between participant differences in the extent of letter identification slowing following moderate (versus strong) threat distractor faces at long distractor-target ISIs was associated with a heightened FFA response and decreased late (FIR bin 4) dACC response to these distractors. This is consistent with engagement of dACC in reactive control being required to overcome stimulus specific activity in FFA, in order to fully re-allocate resources to processing of task-relevant stimuli. More speculatively, ongoing activation coding for faces (as a result of the recently viewed distractor) may have interfered with preparatory activation of representations of the two potential target letters. Such preparatory activation in higher visual regions has indeed been shown to be predictive of faster target identification (Stokes et al., 2009). This could also explain the relationship between the FIR bin 4 dACC response and participants’ speed of letter identification following moderate versus full threat distractor faces at long ISIs. This time bin reflects neuronal activity pertaining to a time point (around and slightly prior to target onset) at which top-down inhibition of distractor-related activity and re-allocation of processing resources to target-related templates is likely to be especially important for fast letter identification performance.

Of note, FFA activity and late (bin 4) dACC activity jointly mediated the relationship between trait anxiety and slowed letter identification following moderate versus full threat distractors at long distractor-target ISIs. Paralleling our findings for behavioral interference (letter identification RT slowing), no anxiety-related differences were observed in brain activity indices at short distractor-target ISIs. On these short ISI trials, the extent of RT slowing after moderate versus full threat distractor faces shown by participants was related negatively to recruitment of dACC (FIR time bin 3, see **Table 3** and **Figure 3**; also FIR time bin 2, see Table S2). These effects were however, independent of levels of trait anxiety. In other words, low and high trait anxious individuals did not differ in the extent to which they were disrupted by distractors of moderate threat value at the shorter ISIs, consistent with the effects of anxiety primarily being upon recovery from rather than initial response to such stimuli.

Trait anxiety was also positively associated with increased amygdala activity to moderate versus strong threat distractor faces at long distractor-target ISIs. Indeed this relationship was rather more robust than the relationship between anxiety and FFA activity for this contrast, being observed bilaterally and surviving correction for across hemisphere multiple comparisons. Interestingly, our analyses did not reveal any direct mediating role of amygdala activity in the relationship between anxiety and slowed letter identification following moderate versus strong threat distractor faces. However, there was a significant relationship, across participants, between the magnitude of the amygdala response and the extent of FFA activity, the latter in turn playing a direct role in mediating the anxiety – letter identification RT interference relationship. One possible interpretation of these findings is that while the amygdala acts as an initial trigger indicating the presence of potential threat (LeDoux, 2000), it is recurrent processing in stimulus specific areas responsive to the distractor stimulus – FFA in the context of the present study – that determines whether attentional interference is observed seconds after distractor termination. This is in line with suggestions by Lim et al. (2009) that when a stimulus (in their case the target rather than a distractor) is of potential threat relevance, the amygdala acts to modulate activity in stimulus specific regions with it being activity in the latter regions that most directly influences task performance. In the case of our current study, the precise role of FFA versus amygdala activity in mediating the relationship between anxiety and prolonged attentional interference following face distractors of moderate threat value should be interpreted with caution given the limited power of our sample size, particularly to detect mediation effects (Fritz and MacKinnon, 2007). Here, replication of the work reported using a larger sample size and collecting more data per subject to increase power for both between and within subjects analyses would be of value. In particular, this would facilitate investigation of whether changes in activity linked to within-subject performance variability involve the same regions as those implicated in anxiety-related between-subject differences in RT slowing following moderate threat distractors.

Our findings extend previous research by indicating that representations of task-irrelevant emotional stimuli can compete with task-relevant processing even when their presentation does not directly coincide with any aspect of task performance. Interfering effects of emotional stimuli, beyond the point that they disappear, have previously been studied using an emotional variant of the attentional blink paradigm. In these studies, presentation of task-irrelevant negatively valenced stimuli has been found to impact upon subsequent target detection accuracy at distractor–target ISIs of ∼200 ms (Most et al., 2005). Such “emotion-induced blindness” effects appear to be particularly associated with threat, rather than other negative stimuli (Vermeulen et al., 2009). Our current results reveal that emotional interference can continue over a considerably longer time course than observed in the context of the emotional blink. Here, it is important to note that emotional blink studies assess failure to detect target stimuli. The effects of processing competition on slowed target identification, as studied here, may be more subtle and observable after longer intervals post distractor offset. These effects may also potentially reflect disruption to a different aspect of perceptual or attentional processing. It has been suggested that emotional blink effects primarily reflect disruption at an early perceptual stage of processing as opposed to a later central stage of competition for attentional resources (Most and Wang, 2011); though it has also been recognized that an interplay of effects at both stages could be present (Wang et al., 2012). It seems plausible that the processing interference reported in the current study involves competition for stimulus representation in working memory, with distractor representations potentially competing with activation of templates for the two possible letter string targets. Whether this is indeed the case will require further studies to determine.

Within the present study, modulation by anxiety of attentional interference effects emerged only when targets followed moderate, potentially ambiguous (surprise/fear blend) threat distractors (versus stronger, less ambiguous (100% fear) threat distractors) and only at the long ISIs (∼3 s), not at the shorter ones (∼1.6 s). This contrasts with previous findings that anxiety modulates the magnitude of spatial attentional interference effects from strong threat distractors (e.g., fearful faces) presented at 0–500 ms prior to target stimuli (e.g., MacLeod and Mathews, 1988; Fox et al., 2001). This difference in findings could reflect differences in the time course of interference from stronger versus more mild threat cues, with our longer ISI being more sensitive to detect the latter and the very short ISIs used in previous studies being more sensitive to detect the former. (Indeed, dot probe studies have reported that anxiety-related spatial attentional capture by strong threat cues is no longer observed if ISIs of ∼1 s are used, e.g., Koster et al., 2005). Such a notion would be consistent with recent suggestions from human and rodent fear conditioning studies that distinct circuitry may underlie initial phasic fear responses to cues of certain threat value versus more prolonged responses when threat is uncertain (Davis et al., 2010), with the latter held to especially characterize patients with anxiety disorders. As ambiguous stimuli involve uncertainty regarding the presence of threat (cf. Grupe and Nitschke, 2013) it seems plausible that such stimuli may also cause a more sustained response than stronger threat stimuli, particularly among anxious individuals. A valuable avenue for future research would be to directly compare interference from strong, unambiguous, versus moderate, potentially ambiguous, threat distractors presented at very short (<500 ms) versus long (≥3 s) intervals prior to targets, ideally both with and without manipulations of spatial attention (see Most and Wang, 2011 for discussion of differing temporal attentional interference effects for common versus distinct spatial locations). For now we note that our study may be the first to reveal trait anxiety-related attentional interference effects associated with a slow sustained “anxiety” response to uncertain threat, as opposed to a fast phasic “fear” response to clear threat signals, with this distinction possibly mapping, at least in part, onto that between recovery and reactivity periods of stimulus response outlined elsewhere (Schuyler et al., 2014).

In summary, our findings provide evidence that, even beyond their offset, representations of task-irrelevant stimuli of potential threat value can compete with those of task-relevant stimuli. This may have the effect of preventing conscious perception of targets altogether at very short SOAs (e.g., in the emotional blink task) and slowing the speed with which task-relevant stimuli can be identified at longer SOAs, as reported here. In the current study, high trait anxious individuals diverged from low trait anxious individuals in showing slowed target identification several seconds after presentation of distractors of moderate, potentially ambiguous, threat value. This was linked to increased distractor-related activity in stimulus specific regions coupled with poorer recruitment of reactive control mechanisms.

These findings have implications for understanding how threat-related attentional biases might result in functional impairments for anxious individuals in daily life. Attentional prioritization of clearly threatening stimuli is not in itself maladaptive and such threatening stimuli may be encountered fairly infrequently. In contrast, milder threat cues may be encountered relatively often, and are less likely to demand an urgent response. They may even represent a potentially benign event, as when dishes clattering to the floor result in an expression of shock, and so may possess a degree of ambiguity as action cues – not clearly promoting a fight/flight response. The failure of trait anxious individuals to rapidly evaluate and terminate processing of such relatively weak, potentially ambiguous, threat cues might lead to an inappropriate long-lasting allocation of resources to a wide range of task-irrelevant stimuli, with resulting decrement to task performance. This could have negative consequences in various social and occupational daily life settings. For example, in the case of driving, disruption for the time it takes a “near miss” to occur may not lead to an accident, but attention being taken off the road for several seconds following this could be a different case altogether. Equally, dwelling on fleeting facial expressions may lead to difficulties with social interactions due to important subsequent conversational content or non-verbal cues being missed. We hope that highlighting how relatively mild, potentially ambiguous, threat stimuli can lead to longer lasting post-offset disruption of processing in high trait anxious individuals, and clarifying the brain mechanisms that may play a role in this, will be of value to both basic and applied research.

## Conflict of Interest Statement

The authors declare that the research was conducted in the absence of any commercial or financial relationships that could be construed as a potential conflict of interest.
