# Supplementary material for: Moderate threat causes longer lasting disruption to processing in anxious individuals
Source: Front Hum Neurosci. 2014 Aug 19;8:626. doi: 10.3389/fnhum.2014.00626 (PMC4137542; doi:10.3389/fnhum.2014.00626)
Supplement: Supplementary file 1 [file Data_Sheet_1.PDF]

25th February 2014

Supplementary Material

Moderate threat causes longer lasting disruption to processing in anxious individuals

Sophie Forster, Anwar O. Nunez-Elizalde, Elizabeth Castle and Sonia J. Bishop

Department of Psychology and Helen Wills Neuroscience Institute, University of California, Berkeley, CA, USA

*** Correspondence:** Sonia Bishop, Department of Psychology and Helen Wills Neuroscience Institute, 3210 Tolman Hall, University of California, Berkeley, California, 94720-1650, USA

sbishop@berkeley.edu

1. Supplementary Data

Additional regression analyses were conducted to test the robustness of the regression results reported in the main manuscript (**see Table 1**). There, we examined influences of activity in our ROIs (right and left amygdala, right and left FFA, and dACC) on target identification times following moderate versus strong threat distractors - this was tested separately for long and short ISI trials using a step-wise entry method. FFA and amygdala predictors were derived from the main canonical HRF model. DACC activation indices were derived from an additional FIR model employed to capture dACC recruitment at around and slightly preceding tine of target presentation. Allowing for the haemodynamic delay in the BOLD response, this corresponded to FIR time bin 3 (4-6s post distractor onset) for short ISI trials and FIR time bin 4 (6-8s post distractor onset) for long ISI trials.

In supplementary analyses, we first confirmed that using a Forced Entry regression model with the same set of predictors as the step-wise model reported in **Table 1a** (canonical indices of left and right amygdala and FFA activity, and FIR time bin 4 dACC activity to moderate versus strong threat distractors, for long distractor-target ISI trials) replicated the results reported in **Table 1a.** Specifically, the overall model was significant ($r^2 = .77$, $p < .001$); and, as in our step-wise model, only bin 4 dACC ($\beta = -.58$, $p < .01$) and left FFA ($\beta = .75$, $p < .001$) contributed significantly to the prediction of RT slowing following moderate versus strong threat distractors. We next examined whether our regression findings were robust to replacement of our canonical model indices of FFA and amygdala activity with indices from the FIR model. To this end, we conducted stepwise regression analyses entering FIR time bins 3 and 4 for each of the ROIs (left and right FFA, left and right amygdala, and dACC) as predictors of RT interference. As can be seen in **Table S1**, this replicated the results reported in the main manuscript. Specifically, at long distractor-target ISIs, time bin 3 left FFA activity (the closest to the peak of the canonical HRF), positively, and time bin 4 dACC activity, negatively, emerged as independent and additive predictors of RT interference following moderate versus strong threat distractor faces (**Table S1a**). As in the analyses reported in the main text, although amygdala activity did not emerge as a primary predictor of RT interference, a correlation was observed between FIR time bin 3 amygdala and left FFA activity, $r(20) = .47$, $p < .05$. Meanwhile, also replicating the findings reported in the main manuscript, for short distractor-target ISI trials, time bin 3 dACC activity emerged as the sole predictor of letter string identification time following moderate versus strong threat distractors (**Table S1b**). For the interested reader we have also included the results of a parallel analysis including time bin 2 and well as bins 3 and 4

(**Table S2**). Here, time bin 2 dACC replaces time bin 3 dACC as a predictor of letter string identification time following moderate versus strong threat distractors for short distractor-target ISI trials. Neither of these short ISI dACC activation indices was significantly associated with trait anxiety.

2. Supplementary Figures and Table

2.1. Supplementary Tables

Supplementary Table S1. Results of regression analyses examining brain activity predictors of letter identification slowing (reaction time cost) following moderate fear versus full fear face distractors, using FIR model activation indices for all regions for time bins 3-4 and stepwise entry of predictors

**(a) long distractor-target ISI trials:**					
Predictors	β	P	R^2 for model	Adj. R^2	P for model
dACC (time bin 4)<br>left FFA (time bin 3)	-.59<br>.40	.002<br>.024	.54	.49	<.01
**(b) short distractor-target ISI trials**					
Predictors	β	P	R^2 for model	Adj. R^2	P for model
dACC (time bin 3)	-.47	.033	.22	.18	<.05

Note. FIR = Finite Impulse Response. Time bins 3 and 4 = 4-6s and 6-8s post distractor onset, respectively. dACC = Dorsal Anterior Cingulate Cortex. FFA = Fusiform Face Area

Supplementary Table S2. Results of regression analyses examining brain activity predictors of letter identification slowing (reaction time cost) following moderate fear versus full fear face distractors, using FIR model activation indices for all regions for time bins 2-4 and stepwise entry of predictors

**(a) long distractor-target ISI trials:**					
Predictors	β	P	R^2 for model	Adj. R^2	P for model
dACC (time bin 4)<br>left FFA (time bin 3)	-.59<br>.40	.002<br>.024	.54	.49	<.01
**(b) short distractor-target ISI trials**					
Predictors	β	P	R^2 for model	Adj. R^2	P for model
dACC (time bin 2)	-.54	.011	.29	.25	<.025

Note. FIR = Finite Impulse Response. Time bins 2, 3 and 4 = 2-4s, 4-6s and 6-8s post distractor onset, respectively. dACC = Dorsal Anterior Cingulate Cortex. FFA = Fusiform Face Area.

2.2. Supplementary Figures

Supplementary Figure 1. Regions of Interest (ROIs). The ROIs for left and right FFA (A) were 8mm spheres centred on peak activations taken from task data described in Bishop et al., (2004b), MNI co-ordinates, right: 42, -52, -20; left: -40, -50, -18. The amygdala ROIs (B) were taken from the MNI Automated Anatomical Labeling (AAL) atlas. The dACC ROI (C) was a 10mm radius sphere centered on MNI coordinates: 0, 30, 21. This central co-ordinate was derived from a meta-analysis of cognitive control tasks (Duncan & Owens, 2000) as described previously (Bishop et al., 2008, Forster et al., 2013).

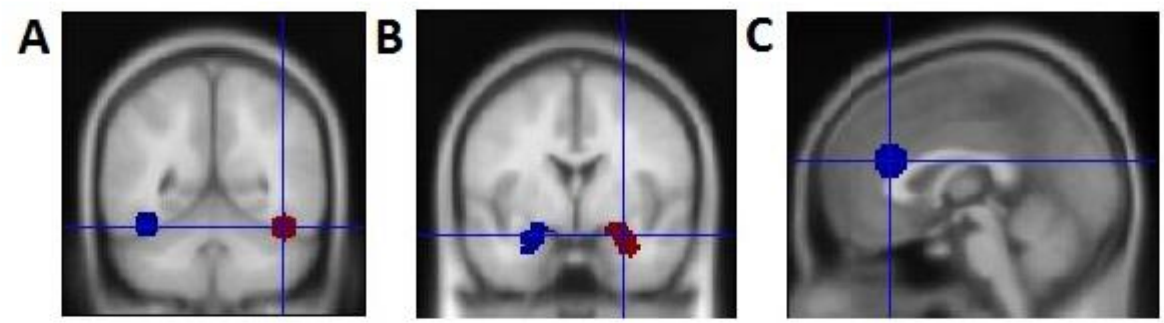

Supplementary Figure 2. Finite impulse response (FIR) waveforms by distractor type plotted separately as a function of anxiety group (median split: low, high) and ISI (short,long). Here the mean activation (against baseline) for each FIR time bin is plotted for each region of interest. Please note that these plots are provided for illustrative purposes only, upon reviewer request. Activity against implicit baseline for a fast event-related design is inevitably noisy hence the large standard errors. Using a median split, some medium level anxious individuals will be included in each group also adding to the noise around the high and low anxiety group means.

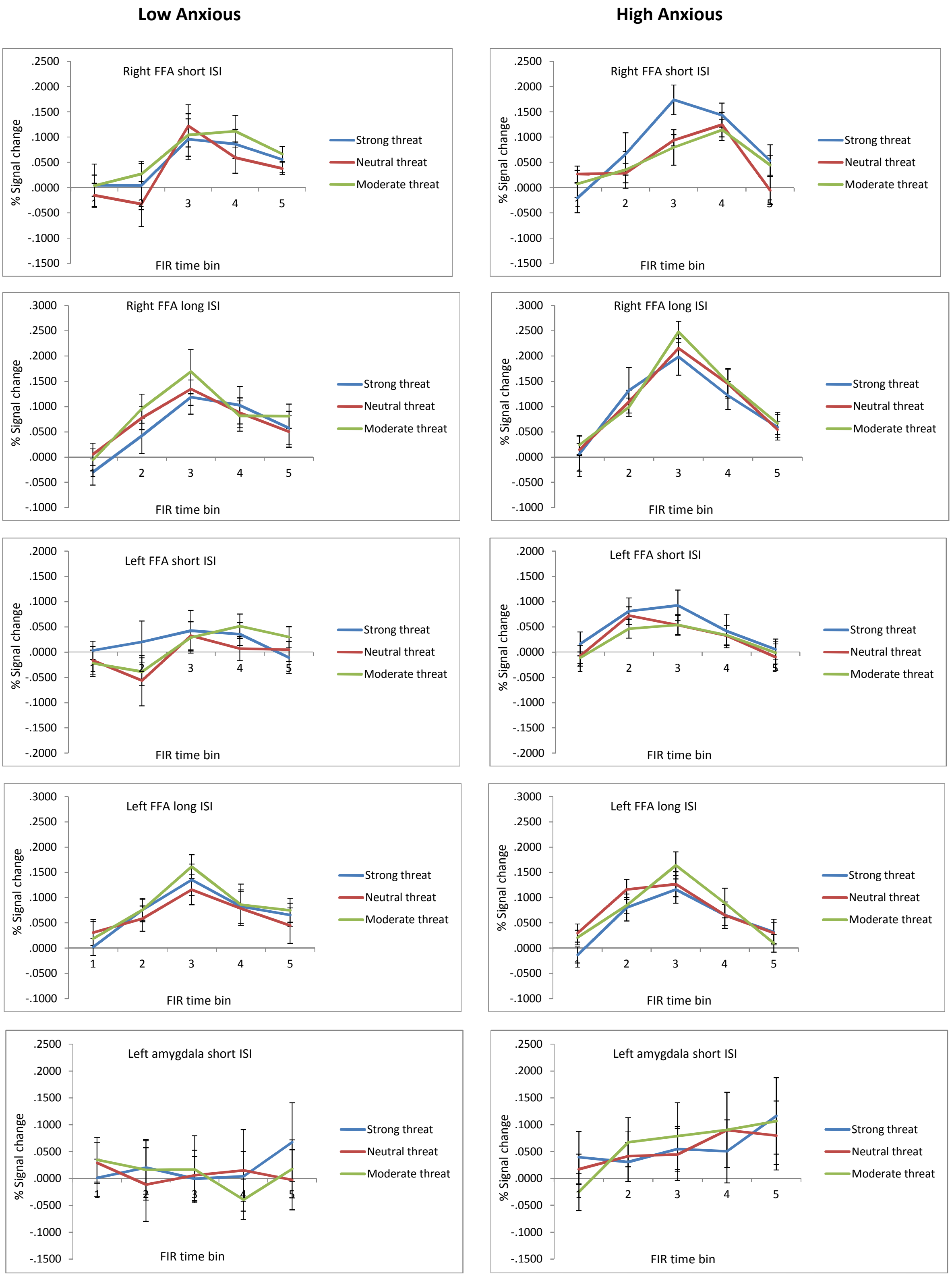

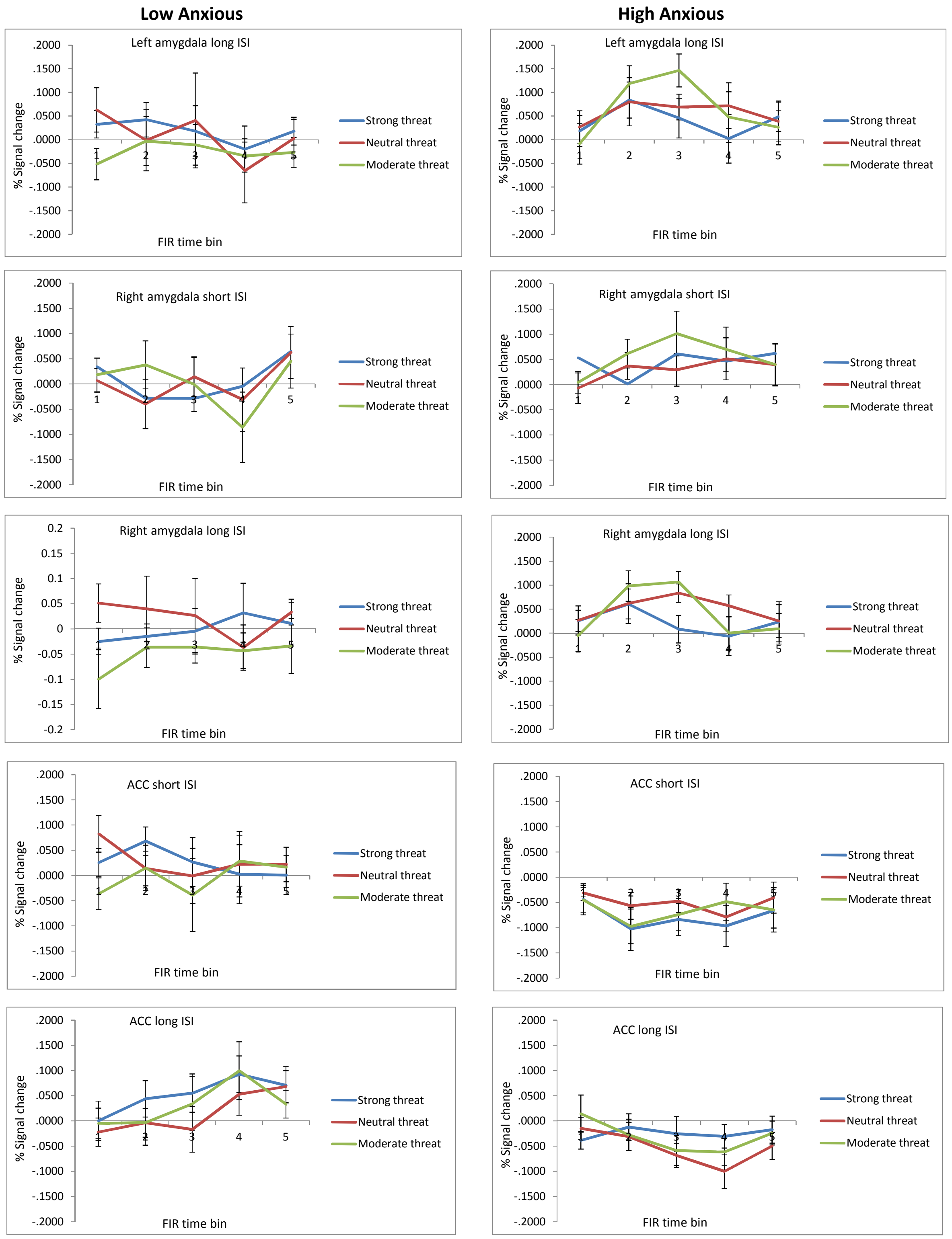

Low Anxious
High Anxious
Left amygdala long ISI
Right amygdala short ISI
Right amygdala long ISI
ACC short ISI
ACC long ISI
% Signal change
FIR time bin
Strong threat
Neutral threat
Moderate threat
